# Supplementary material for: Phase Angle Is a Stronger Predictor of Hospital Outcome than Subjective Global Assessment—Results from the Prospective Dessau Hospital Malnutrition Study
Source: Nutrients. 2022 Apr 24;14(9):1780. doi: 10.3390/nu14091780 (PMC9100773; doi:10.3390/nu14091780)
Supplement: Supplementary file 1 [file nutrients-14-01780-s001.zip › nutrients-1693970-supplementary.pdf]

Table S1. Cox cause specific proportional hazards analysis.

		Model I	Model II	Model III
Variable	N	Estimate + 95% CI	Estimate + 95% CI	Estimate + 95% CI
SGA—A	584 (39%)	1.00	1.00	1.00
SGA—B/C	915 (61%)	0.69 (0.62, 0.78) ***	0.9 (0.77, 1.06)	0.94 (0.78, 1.13)
Phase angle ≤3	327 (22%)		0.43 (0.35, 0.52) ***	0.56 (0.45, 0.7) ***
Phase angle 3–4	482 (32%)		0.61 (0.49, 0.77) ***	0.77 (0.6, 0.99) #
Phase angle 4–5	424 (28%)		0.73 (0.63, 0.86) ***	0.8 (0.68, 0.94) *
Phase angle >5	272 (18%)		1.00	1.00
Age ≤65	375 (25%)	0.93 (0.72, 1.2)	0.8 (0.63, 1.01)	0.81 (0.65, 1.02)
Age 65-75	361 (24%)	1.00	1.00	1.00
Age 75–80	337 (22%)	1 (0.83, 1.2)	0.98 (0.83, 1.17)	0.97 (0.84, 1.12)
Age >80	432 (29%)	1.09 (0.93, 1.28)	1.16 (0.98, 1.36)	1.11 (0.95, 1.29)
BMI ≤18.5	162 (11%)	1.06 (0.86, 1.3)	1.13 (0.95, 1.35)	1.1 (0.89, 1.34)
BMI 18.5–25	715 (48%)	1.00	1.00	1.00
BMI 25–30	379 (25%)	0.95 (0.79, 1.14)	0.96 (0.79, 1.18)	1.01 (0.82, 1.24)
BMI >30	241 (16%)	0.74 (0.61, 0.89) *	0.76 (0.62, 0.95) #	0.82 (0.66, 1.02)
CRP ≤10	480 (32%)			1.00
CRP 10–100	673 (45%)			0.73 (0.61, 0.86) **
CRP >100	158 (10%)			0.58 (0.47, 0.71) ***
CRP -no value	194 (13%)			1.97 (1.47, 2.66) ***
Sex—male	723 (48%)	1.00	1.00	1.00
Sex—female	782 (52%)	1.02 (0.94, 1.11)	1.07 (0.99, 1.16)	0.97 (0.89, 1.07)
ICD A–B: infections	79 (5%)	0.87 (0.71, 1.07)	0.92 (0.74, 1.14)	0.92 (0.72, 1.16)
ICD C–D50: neoplasms	356 (24%)	0.62 (0.46, 0.85) *	0.62 (0.45, 0.85) *	0.61 (0.45, 0.84) *
ICD D50–89: blood and blood forming organs	72 (5%)	1.16 (0.9, 1.51)	1.26 (0.97, 1.62)	1.15 (0.92, 1.44)
ICD E: endocrine	35 (2%)	1.36 (0.78, 2.39)	1.31 (0.69, 2.5)	1.18 (0.64, 2.17)
ICD G: nervous system	39 (3%)	1.05 (0.71, 1.55)	1.06 (0.71, 1.58)	0.8 (0.48, 1.33)
ICD I: circulatory	176 (12%)	0.62 (0.49 ,0.79) ***	0.61 (0.47, 0.79) **	0.53 (0.4, 0.7) ***
ICD J: respiratory	110 (7%)	0.72 (0.52, 1.01)	0.71 (0.51, 0.98) #	0.69 (0.52, 0.92) #
ICD 1 K: digestive	286 (19%)	1.00	1.00	1.00
ICD M: mouskoskeletal	60 (4%)	0.54 (0.38, 0.76) **	0.54 (0.38, 0.77) **	0.49 (0.35, 0.69) ***
ICD N: genitourinary	62 (4%)	1.04 (0.68, 1.57)	1.1 (0.72, 1.67)	1.14 (0.88, 1.48)
ICD other	37 (2%)	0.9 (0.62, 1.31)	0.87 (0.59, 1.27)	0.78 (0.53, 1.15)
ICD R: abnormal findings	65 (4%)	1.36 (0.89, 2.08)	1.32 (0.84, 2.08)	1.21 (0.8, 1.83)
ICD S–T: injury, poison	103 (7%)	0.47 (0.37, 0.59) ***	0.48 (0.38, 0.59) ***	0.49 (0.41, 0.58) ***
**CONCORDANCE**		**0.616**	**0.639**	**0.672**

Model I: Age, sex, BMI, ICD-10 disease category and SGA. Model II: PhA added to model I. Model III: CRP added to model II. All data are hazard ratios (HR) and 95% confidence intervals (in parentheses). # $p < 0.05$, * $p < 0.01$, ** $p < 0.001$, *** $p < 0.0001$. BMI, body mass index; ICD-10, 10th revision of the International Statistical Classification of Diseases and Related Health Problems; ICD A–T, disease classification; SGA, Subjective Global Assessment; SGA A, B and C, types of nutrition assessment; PhA, Phase Angle; CRP, C-reactive protein; CI, confidence interval.

Table S2. Main Fine and Gray analysis excluding readmissions.

		Model I	Model II	Model III
Variable	N	Estimate + 95%CI	Estimate + 95%CI	Estimate + 95%CI
SGA—A	522 (42%)	1.00	1.00	1.00
SGA—B/C	733 (58%)	0.72 (0.67, 0.77) ***	0.91 (0.81, 1.03)	0.94 (0.82, 1.09)
Phase angle ≤3	254 (20%)		0.46 (0.37, 0.59) ***	0.59 (0.44, 0.78) **
Phase angle 3–4	398 (32%)		0.68 (0.57, 0.82) ***	0.84 (0.68, 1.03)
Phase angle 4–5	358 (28%)		0.8 (0.68, 0.93) **	0.85 (0.72,1) #
Phase angle >5	251 (20%)		1.00	1.00
Age ≤65	318 (25%)	0.84 (0.67, 1.06)	0.73 (0.6, 0.89) *	0.74 (0.61, 0.9) *
Age 65–75	286 (23%)	1.00	1.00	1.00
Age 75–80	280 (22%)	0.87 (0.7, 1.07)	0.84 (0.68, 1.04)	0.84 (0.68, 1.03)
Age >80	377 (30%)	0.93 (0.81, 1.07)	0.98 (0.84, 1.14)	0.93 (0.79, 1.09)
BMI ≤18.5	125 (10%)	1 (0.89, 1.12)	1.02 (0.89, 1.16)	0.94 (0.79, 1.11)
BMI 18.5-25	584 (47%)	1.00	1.00	1.00
BMI 25–30	332 (26%)	1 (0.86, 1.15)	1 (0.86, 1.17)	1.06 (0.91, 1.24)
BMI >30	213 (17%)	0.84 (0.73, 0.96)#	0.86 (0.73, 1.01)	0.9 (0.79, 1.03)
CRP ≤10	411 (33%)			1.00
CRP 10–100	547 (43%)			0.74 (0.6, 0.9) *
CRP >100	132 (10%)			0.55 (0.43, 0.7) ***
CRP—no value	171 (14%)			1.7 (1.23, 2.35) *
Sex—male	598 (47%)	1.00	1.00	1.00
Sex—female	663 (53%)	1.04 (0.97, 1.12)	1.08 (1, 1.17) #	0.96 (0.86, 1.08)
ICD A–B: infections	65 (5%)	0.84 (0.69,1.02)	0.88 (0.73,1.05)	0.87 (0.69,1.1)
ICD C–D50: neoplasms	271 (22%)	0.61 (0.46 ,0.79) **	0.6 (0.46, 0.8) **	0.58 (0.44, 0.76) ***
ICD D50–89: blood and blood forming organs	62 (5%)	1.09 (0.78, 1.54)	1.11 (0.78, 1.57)	0.96 (0.64, 1.43)
ICD E: endocrine	33 (3%)	1.51 (0.83, 2.74)	1.41 (0.7, 2.84)	1.29 (0.67, 2.51)
ICD G: nervous system	36 (3%)	1.14 (0.85, 1.54)	1.12 (0.81, 1.55)	0.85 (0.54, 1.35)
ICD I: circulatory	156 (12%)	0.58 (0.48, 0.71) ***	0.57 (0.46, 0.71) ***	0.51 (0.39, 0.66) ***
ICD J: respiratory	89 (7%)	0.61 (0.48, 0.78) ***	0.61 (0.48, 0.78) ***	0.61 (0.5, 0.75) ***
ICD 1 K: digestive	244 (20%)	1.00	1.00	1.00
ICD M: mouskoskeletal	55 (4%)	0.66 (0.48, 0.91) #	0.65 (0.47, 0.91) #	0.63 (0.45, 0.88) *
ICD N: genitourinary	58 (5%)	1 (0.72, 1.39)	1.03 (0.73, 1.46)	1.1 (0.88, 1.38)
ICD other	31 (2%)	0.65 (0.38, 1.09)	0.57 (0.32, 0.99) #	0.49 (0.27, 0.87) #
ICD R: abnormal findings	58 (5%)	1.25 (0.74, 2.11)	1.23 (0.7, 2.18)	1.03 (0.59, 1.81)
ICD S–T: injury, poison	91 (7%)	0.54 (0.44, 0.65) ***	0.56 (0.47, 0.66) ***	0.57 (0.49, 0.67) ***
**CONCORDANCE**		**0.614**	**0.636**	**0.666**

Model I: Age, sex, BMI, ICD-10 disease category and SGA. Model II: PhA added to model I. Model III: CRP added to model II. All data are hazard ratios (HR) and 95% confidence intervals (in parentheses). # $p < 0.05$, * $p < 0.01$, ** $p < 0.001$, *** $p < 0.0001$.

Table S3. Fine and Gray analysis including Pandora variables and weightloss.

		Model I	Model II	Model III
	n (%)	Estimate + 95% CI	Estimate + 95% CI	Estimate + 95% CI
SGA—A	584 (39%)	1.00	1.00	1.00
SGA—B/C	915 (61%)	0.85 (0.78, 0.92) ***	0.99 (0.89, 1.09)	1.03 (0.91, 1.15)
Phase angle ≤3	327 (22%)		0.59 (0.47, 0.74) ***	0.72 (0.57, 0.91) *
Phase angle 3–4	482 (32%)		0.73 (0.62, 0.87) **	0.88 (0.74, 1.05)
Phase angle 4–5	424 (28%)		0.87 (0.77, 0.99) #	0.9 (0.79, 1.02)
Phase angle >5	272 (18%)		1.00	1.00
Age ≤65	375 (25%)	0.86 (0.71, 1.05)	0.79 (0.66, 0.95) #	0.79 (0.66, 0.94) *
Age 65–75	361 (24%)	1.00	1.00	1.00
Age 75–80	337 (22%)	0.91 (0.76, 1.09)	0.91 (0.76, 1.08)	0.92 (0.78, 1.08)
Age >80	432 (29%)	1.01 (0.87, 1.16)	1.05 (0.91, 1.21)	0.99 (0.87, 1.14)
BMI ≤18.5	162 (11%)	0.99 (0.83, 1.17)	1.03 (0.89, 1.21)	0.99 (0.81, 1.2)
BMI 18.5–25	715 (48%)	1.00	1.00	1.00
BMI 25–30	379 (25%)	1.09 (0.93, 1.28)	1.06 (0.9, 1.25)	1.11 (0.94, 1.33)
BMI >30	241 (16%)	0.99 (0.9, 1.09)	0.94 (0.85, 1.04)	1.01 (0.89, 1.15)
Can you walk? Yes	859 (57%)	1.00	1.00	1.00
Can you walk? With assistance	432 (29%)	0.79 (0.64, 0.99)	0.84 (0.67, 1.05)	0.85 (0.67, 1.08)
Can you walk? Stay in bed	203 (14%)	0.59 (0.48, 0.73) ***	0.66 (0.52, 0.83) **	0.68 (0.55, 0.86) **
CRP ≤10	480 (32%)			1.00
CRP 10–100	673 (45%)			0.74 (0.63, 0.88) **
CRP >100	158 (10%)			0.54 (0.42, 0.7) ***
CRP—missing	194 (13%)			1.74 (1.27, 2.39) **
Eaten today—all	933 (62%)	1.00	1.00	1.00
Eaten today—half	262 (18%)	0.75 (0.64, 0.89) **	0.77 (0.66, 0.91) *	0.83 (0.72, 0.97) #
Eaten today—quarter	49 (3%)	0.7 (0.51, 0.96) #	0.74 (0.52, 1.05)	0.84 (0.61, 1.17)
Eaten today—nothing, allowed	28 (2%)	0.85 (0.52, 1.4)	0.85 (0.53, 1.36)	0.87 (0.52, 1.45)
Eaten today—nothing, not allowed	223 (15%)	1.22 (1.04, 1.44) #	1.19 (1.01, 1.39) #	1.27 (1.07,1.51) *
Fluid status—normal	826 (55%)	1.00	1.00	1.00
Fluid status—overloaded	374 (25%)	0.94 (0.89,1)#	1.03 (0.95,1.1)	1.03 (0.94,1.13)
Fluid status—dry	295 (20%)	1.08 (0.91,1.28)	1.02 (0.87,1.19)	1 (0.88,1.13)
Sex—male	723 (48%)	1.00	1.00	1.00
Sex—female	782 (52%)	1.08 (0.96 ,1.2)	1.1 (0.99, 1.22)	1 (0.9, 1.11)
ICD A–B: infections	79 (5%)	0.96 (0.81, 1.14)	0.96 (0.82, 1.13)	0.97 (0.81, 1.17)
ICD C–D50: neoplasms	356 (24%)	0.63 (0.48, 0.82) **	0.62 (0.47,0 .82) **	0.61 (0.47, 0.79) **
ICD D50–89: blood and blood forming organs	72 (5%)	1.16 (0.83, 1.63)	1.18 (0.83, 1.66)	1.08 (0.79, 1.47)
ICD E: endocrine	35 (2%)	1.45 (0.83, 2.55)	1.39 (0.76, 2.52)	1.28 (0.73, 2.24)
ICD G: nervous system	39 (3%)	1.35 (0.97, 1.89)	1.27 (0.93, 1.74)	0.97 (0.65, 1.46)
ICD I: circulatory	176 (12%)	0.66 (0.57, 0.77) ***	0.64 (0.53, 0.76) ***	0.57 (0.46, 0.71) ***
ICD J: respiratory	110 (7%)	0.68 (0.53, 0.87) *	0.65 (0.5, 0.85) *	0.64 (0.51, 0.8) ***
ICD K: digestive	286 (19%)	1.00	1.00	1.00
ICD M: mouskoskeletal	60 (4%)	0.72 (0.49, 1.08)	0.68 (0.46, 1.02)	0.63 (0.42, 0.94) #
ICD N: genitourinary	62 (4%)	0.95 (0.65, 1.4)	0.97 (0.65, 1.44)	1.03 (0.78, 1.35)
ICD other	37 (2%)	0.71 (0.42, 1.19)	0.64 (0.38, 1.09)	0.56 (0.31, 1)
ICD R: abnormal findings	65 (4%)	1.15 (0.67, 1.98)	1.09 (0.63, 1.88)	0.96 (0.56, 1.65)
ICD S–T: injury, poison	103 (7%)	0.62 (0.52, 0.76) ***	0.61 (0.5, 0.74) ***	0.63 (0.53, 0.74) ***
Weight loss <0%	208 (14%)	0.9 (0.77, 1.04)	0.92 (0.79, 1.07)	0.86 (0.74, 1) #
Weight loss 0%	259 (18%)	1.15 (1, 1.33)	1.16 (1.01, 1.34) #	1.14 (0.99, 1.32)
Weight loss 0–5%	312 (22%)	1.00	1.00	1.00
Weight loss 5–10%	309 (21%)	1.04 (0.9, 1.2)	1.05 (0.91, 1.21)	1.03 (0.89, 1.2)

Weight loss >10%	352 (24%)	1 (0.88, 1.13)	1.04 (0.92, 1.16)	1.07 (0.95, 1.21)
**CONCORDANCE**		**0.631**	**0.639**	**0.666**

Model I: Age, sex, BMI, can you walk, eaten today, fluid status, ICD-10 disease category, SGA and weight loss. Model II: PhA added to model I. Model III: CRP added to model II. All data are hazard ratios (HR) and 95% confidence intervals (in parentheses). # $p < 0.05$, * $p < 0.01$, ** $p < 0.001$, *** $p < 0.0001$.
